# Malaria Control and Public Health

**DOI:** 10.3201/eid1006.030787

**Published:** 2004-06

**Authors:** Donald Roberts, Christopher Curtis, Richard Tren, Brian Sharp, Clive Shiff, Roger Bate

**Affiliations:** * Uniformed Services University of the Health Sciences, Bethesda, Maryland, USA;; † London School of Hygiene & Tropical Medicine, London, United Kingdom;; ‡ Africa Fighting Malaria, Sandton, South Africa;; § Medical Research Council, Durban, South Africa;; ¶ Johns Hopkins University, Baltimore, Maryland, USA;; # Africa Fighting Malaria, Washington, D.C., USA

**Keywords:** DDT, malaria control, birth rate, lactation, Africa

**To the Editor**: Malaria continues to cause disease and death in millions of persons living in areas of the world where it is endemic, despite 4 decades of research on vaccines, new drugs, and alternative methods of control. Still, by far the most effective method for reducing and controlling the impact of this disease is indoor residual spraying (IRS) of insecticides. The most cost-effective and safe insecticide has been, and in many instances still is, dichlorodiphenyltrichloroethane (DDT). This intervention is continually under scrutiny, and we address these issues in this letter.

Chen and Rogan (*1*) claim that DDT causes reduced duration of lactation and increased incidence of preterm births, and they posit that DDT used for malaria control would do as much harm as good. The validity of their arguments requires substantial evidence of a causal relationship between DDT and adverse consequences of DDT IRS for malaria control.

Chen and Rogan dismiss a field study on births and duration of lactation in South African mothers, some of whom occupied houses sprayed with DDT for malaria control (*2*). However, if claims of large numbers of adverse health effects of DDT IRS are correct, then the study should have detected large differences between DDT-exposed and unexposed populations. According to Chen and Rogan, the median duration of breastfeeding could be as low as 3–4 months when mothers are exposed to high levels of DDT. Thus, a cross-section of breastfeeding infants in the DDT-exposed population should, on average, have been considerably younger than in the unexposed population. In fact, the average age of breastfeeding infants was slightly greater in the DDT-exposed population (8.3 months versus 7.7 months). For both populations, only an insignificant fraction of mothers could not donate milk. Furthermore, twice the level of dichlordiphenylethylene (DDE, metabolic breakdown product of DDT) that is claimed to cause reduced duration of lactation in humans has no adverse affect on lactation in rats (*3*). The authors of the South African study (*2*) report no difference in rates of stillbirths between the sprayed and unsprayed areas.

The National Institute of Environmental Health Sciences study (*4*) reported a causal association between DDT and preterm and small-for-gestational-age births but this has not been replicated for African births. The study was not based on a random population of births, and no explanation is offered for including diverse categories of births in the study population.

An earlier study in Sri Lanka presented data on deaths attributed to malaria and to premature births years before DDT was used and years when DDT IRS was used in 21 districts (*5*). Districts varied greatly in levels of malaria endemicity. After DDT was introduced in 1946, levels of IRS in 21 districts were commensurate with levels of endemic malaria. After 1946, malaria deaths declined greatly and the reduction was greatest where DDT usage was highest. During the same period, deaths attributable to premature births increased slightly. Investigators attributed this to "improvements in reporting and diagnosis rather than any declines in the health of expectant mothers, which on all other criteria showed improvement." (*5*). Spearman's correlation analysis for 21 districts shows that the increase in premature birth deaths was slightly greater in areas with less malaria and DDT use. Thus, the evidence does not support the idea that the reported increase in premature births was a side effect of DDT use. In any case, the increase in deaths attributable to premature births was orders of magnitude less than the reduction in deaths directly caused by malaria and other conditions indirectly related to malaria (*5*).

Similar major benefits of DDT use were seen in Guyana, where in 2 to 3 years, near elimination of malaria halved maternal deaths and reduced infant deaths by 39% (*6*). Anemia-associated deaths in pregnant females were reduced from 10 to 2.3 per 1,000 adult deaths (*7*). There was no offset of infant deaths attributable to adverse effects of DDT. Data from Guyana are particularly relevant to the present issue because malaria control was entirely due to DDT, i.e., drug treatments were not included (*7*). Health improvements related to DDT use accounted for 21% to 56% of increased population growth in Guyana during the postwar years (*5*).

In summary, these data from South Africa, Sri Lanka, and Guyana are clearly contrary to the claims of Chen and Rogan (*1*) that ill effects of DDT on maternal health and infant survival would counterbalance the beneficial effect of malaria control. Their claim that alternative chemicals are cheaper than DDT is incorrect (*8*). Recent data on pyrethroid-treated bed nets are encouraging for situations in which sustained provision of spray pumps and trained spray teams are not feasible. However, even the best results with these nets do not match those obtained in the past with IRS, e.g., the suppression of malaria infection in Zanzibar from holoendemic levels to <5% (*9*).

In recent years, programs in South Africa and Madagascar (*10*) that again started IRS with DDT have greatly reduced malaria and malaria-related deaths. DDT is still needed and research is required to improve its use. The Stockholm Convention on Persistent Organic Pollutants specifically allows continued public health use of DDT.
