# Supplementary material for: Malaria Control and Public Health
Source: Emerg Infect Dis. 2004 Jun;10(6):1170–1. doi: 10.3201/eid1006.030787 (PMC3323159; doi:10.3201/eid1006.030787)
Supplement: Related texts

# Reply: Malaria Control and Public Health

**Authors:** Aimin Chen, Walter J. Rogan

**Affiliations:** * National Institute of Environmental Health Sciences, Research Triangle Park, North Carolina, USA

**In Reply:** We do not believe that causality has been demonstrated for the relationship between dichlordiphenylethylene (DDE) and shorter period of lactation or preterm birth. However, we think the evidence is sufficiently strong that the possibility of causality cannot be dismissed and testing this hypothesis will require data from appropriately designed studies in areas where dichlorodiphenyltrichloroethane (DDT) is used.

We think that the cross-sectional study (*1*) referred to by Roberts et al. (*2*) cannot determine whether DDE shortens lactation. Women with higher levels of DDE and shorter lactation periods would be less likely than women with lower levels of DDE and longer lactation periods to appear in such a study, which would mask any associations.

As noted in the Longnecker report on the association between DDE and preterm birth (*3*), several previous studies have shown such an effect, but they were relatively small. That the perinatal collaborative study was not a random sample of U.S. births does not seem relevant. Women could not choose whether to participate on the basis of their DDT level because they did not know it and could not choose whether to participate on the basis of a preterm birth because they were enrolled during pregnancy.

Roberts et al. reference success stories of DDT use from the 1930s to the 1950s in Sri Lanka and Guyana. The reports of the Sri Lanka study are not in the peer-reviewed literature. Data from the Guyana sugar plantations must include factors other than DDT and malaria because profound differences existed in all-cause mortality in adults and children over the span of the reports. Whether DDT was effective in those areas at that time cannot determine whether it would be a two-edged sword now.

Although the p,p´ isomer of DDE is in human tissue at the highest concentration, technical DDT contains approximately 10% of the o,p isomer, and o,p-DDE can be detected if sought (*4*). We and others measure p,p´-DDE as an index of total DDE, but our hypothesis for the estrogenic mechanism by which lactation time might be shortened involves the estrogenic isomer o,p-DDE. The Kornbrust study of DDE and lactation in rodents used pure p, p-DDE, the most prevalent congener but also the least estrogenic one (*5*). Since the hypothesis concerned p,p´-DDE, the estrogenic congener, the work was unfortunately noncontributory.

Malaria is a major public health problem, and vigorous efforts to prevent and treat it are necessary. We fear, though, that DDT is not entirely benign and have some evidence to show this. Proceeding as if the safety of DDT had been demonstrated absolutely does not seem a prudent course. DDT is inexpensive; however, cost is irrelevant if DDT use causes as many infant deaths as it prevents.

## References

[1] Bouwman H, Reinecke AJ, Cooppan RM, Becker PJ. Factors affecting levels of DDT and metabolites in human breast milk from Kwazulu. J Toxicol Environ Health. 1990;31:93–115. 10.1080/152873990095314402213927

[2] Roberts D, Curtis C, Tren R, Sharp B, Shiff C, Bate R. Malaria control and public health. Emerg Infect Dis. 2004;10:1170–1.1522467710.3201/eid1006.030787PMC3323159

[3] Longnecker MP, Klebanoff MA, Zhou H, Brock JW. Association between maternal serum concentration of the DDT metabolite DDE and preterm and small-for-gestational-age babies at birth. Lancet. 2001;358:110–4. 10.1016/S0140-6736(01)05329-611463412

[4] Jensen AA. Chemical contaminants in human milk. Residue Rev. 1983;89:1–128.631644110.1007/978-1-4612-5601-4_1

[5] Kornbrust D, Gillis B, Collins B, Goehl T, Gupta B, Schwetz B. Effects of 1,1-dichloro-2,2-bis[p-chlorophenyl]ethylene (DDE) on lactation in rats. J Toxicol Environ Health. 1986;17:23–36. 10.1080/152873986095307993950997
